# Impact of Planting Date on Nutritional Composition of Faba Bean ( *Vicia faba* L.) Seeds Across Varieties

**DOI:** 10.1002/pei3.70117

**Published:** 2026-02-11

**Authors:** Shahram Torabian, Theresa J. Nartea, Salar Farhangi‐Abriz

**Affiliations:** ^1^ Agricultural Research Station, College of Agriculture Virginia State University Petersburg Virginia USA; ^2^ Virginia Cooperative Extension, College of Agriculture Virginia State University Petersburg Virginia USA; ^3^ Cotton Research Institute of Iran, Agricultural Research Education and Extension Organization (AREEO) Gorgan Iran

**Keywords:** calcium concentration, carbohydrate content, phosphorus concentration, protein content, protein‐to‐energy ratio, Ziyad

## Abstract

The legume, Faba bean ( *Vicia faba* L.), offers high nutritional value with consumer market appeal as an affordable plant‐based protein source. Ranking third in global importance after soybean and pea, faba bean provides significant amounts of carbohydrates and essential micronutrients. The purpose of this study is to evaluate the effect of planting date (spring vs. fall) on the seed composition of six faba bean varieties: Grano, Ziyad, Aprovecho, EN3, EN47, and Windsor. Planting date significantly influenced protein and carbohydrate contents as well as the protein‐to‐energy (PE) ratio, but had no significant effect on calorie, ash, or fat content. Fall planting resulted in higher carbohydrate content but lower protein levels and PE ratio compared to spring planting. Among the varieties, Ziyad recorded the highest protein content (26.3%) and the lowest carbohydrate content (60.6%). Additionally, fall planting increased calcium concentration and density, while spring planting enhanced the levels and density of iron, magnesium, and sodium. Potassium content varied significantly among varieties, with EN3 having the lowest (1260 mg/100 g) and Grano the highest (1580 mg/100 g). EN3 (625 mg/100 g) and EN47 (624 mg/100 g) had the lowest phosphorus levels, whereas Ziyad (750 mg/100 g) and Aprovecho (755 mg/100 g) showed the highest. These results highlight the critical role of planting date in determining the nutritional composition of faba bean seeds. Further studies are recommended to investigate amino acid profiles, detailed carbohydrate composition, and fatty acid content across different genotypes and sowing dates.

## 1 Introduction

Rising concerns over excessive consumption of animal‐based proteins linked to chronic health conditions such as obesity, type II diabetes, cardiovascular diseases, and certain cancers prompt the need for healthier and more sustainable protein alternatives. Public scrutiny of the environmental impact of meat production has positioned legumes such as faba bean as a promising solution due to their nutritional, environmental, and economic benefits (Khazaei et al. 2021; Rahate et al. 2021). Among legumes, faba bean ( *Vicia faba* L.), also known as broad bean, stands out as an underutilized but highly nutritious pulse crop. Cultivated globally and ranked as the third most important grain legume (Gu et al. 2020), faba bean offers significant dietary advantages. Its seeds are rich in protein, ranging from 20% to 41% depending on variety, growth conditions, and processing form (Multari et al. 2015; Yang et al. 2018). In addition to protein, faba beans are a valuable source of complex carbohydrates, comprising 51%–68% of the seed weight, with starch being the predominant component (41%–58%) (Dhull et al. 2022; Martineau‐Côté et al. 2022). These carbohydrates provide a slow‐release energy source. The dietary fiber content is also notable, with both soluble and insoluble fractions contributing to total fiber levels of up to 25%. Among legume flours, faba bean flour has been reported to have one of the highest dietary fiber contents (Gu et al. 2020). The faba bean is relatively low in lipids, with total fat content typically less than 2% of seed weight, making it suitable for low‐fat diets. Its lipid profile is composed mainly of unsaturated fatty acids, which are known to support cardiovascular health (Martineau‐Côté et al. 2022). While not a significant energy contributor in terms of fat, this composition adds functional value to its nutritional profile and supports clean‐label food formulation. Minerally, faba bean is a dense source of both macro‐ and micronutrients. It contains substantial levels of potassium (up to 1062 mg/100 g), magnesium, phosphorus, calcium, and iron (Luo et al. 2009; Dhull et al. 2022). The high potassium and low sodium content make faba beans particularly suitable for individuals with hypertension or those following sodium‐restricted diets. Trace elements such as zinc, copper, manganese, and selenium are also present, contributing to immune function, antioxidant defense, and metabolic health.

Faba bean, a cool‐season, nutritionally rich legume, is widely cultivated across the globe (Mínguez and Rubiales 2021). Major producers include Mediterranean countries, Ethiopia, Egypt, China, Afghanistan, India, Northern Europe, and Northern Africa (Rahate et al. 2021). Among the many agronomic factors affecting faba bean performance, sowing date plays a pivotal role in determining not only growth and yield but also the nutritional quality of the harvested seeds (Siddique et al. 2012). The timing of planting can influence the crop's exposure to environmental conditions such as temperature, light, and moisture, all of which directly affect physiological processes and biochemical composition. Our previous research in Virginia, USA, demonstrated that earlier sowing dates, particularly in late September and early October, often result in higher seed yields due to improved vegetative growth and canopy development (Torabian et al. 2024). In contrast, delayed sowing, especially in spring, tends to shorten the growing period, reduce biomass accumulation, and expose plants to terminal stresses such as heat and drought, which can significantly impair productivity (Torabian et al. 2024).

Numerous studies on legumes have shown that planting date has a direct impact on seed nutritional composition, influencing levels of protein, oil, carbohydrates, amino acids, and minerals (El‐Metwally et al. 2007; Bellaloui et al. 2011; Umburanas et al. 2018; Ghadimian et al. 2020; Mostafa et al. 2021; Księżak and Bojarszczuk 2022). For instance, early sowing (e.g., October 1st) has been associated with significant increases in seed protein and carbohydrate content compared to sowing on November 1st (Mostafa et al. 2021). Similarly, sowing on November 25th produced seeds with higher protein content (31.54%) compared to later planting on December 15th (25.90%) (El‐Metwally et al. 2007). In the Nubaria 1 variety, November 25th sowing led to the highest protein content recorded (Shaban et al. 2013).

In the present study, we aim to investigate how two distinct planting windows, fall and spring, affect the nutritional composition of faba bean seeds grown under Virginia conditions. We hypothesize that planting date, by altering physiological and developmental dynamics, will significantly influence key nutritional parameters such as protein, carbohydrates, and minerals. Additionally, we seek to understand varietal responses to planting time, identifying which nutritional components are most sensitive to sowing date and which remain stable across seasons.

## 2 Materials and Methods

**FIGURE 1:**
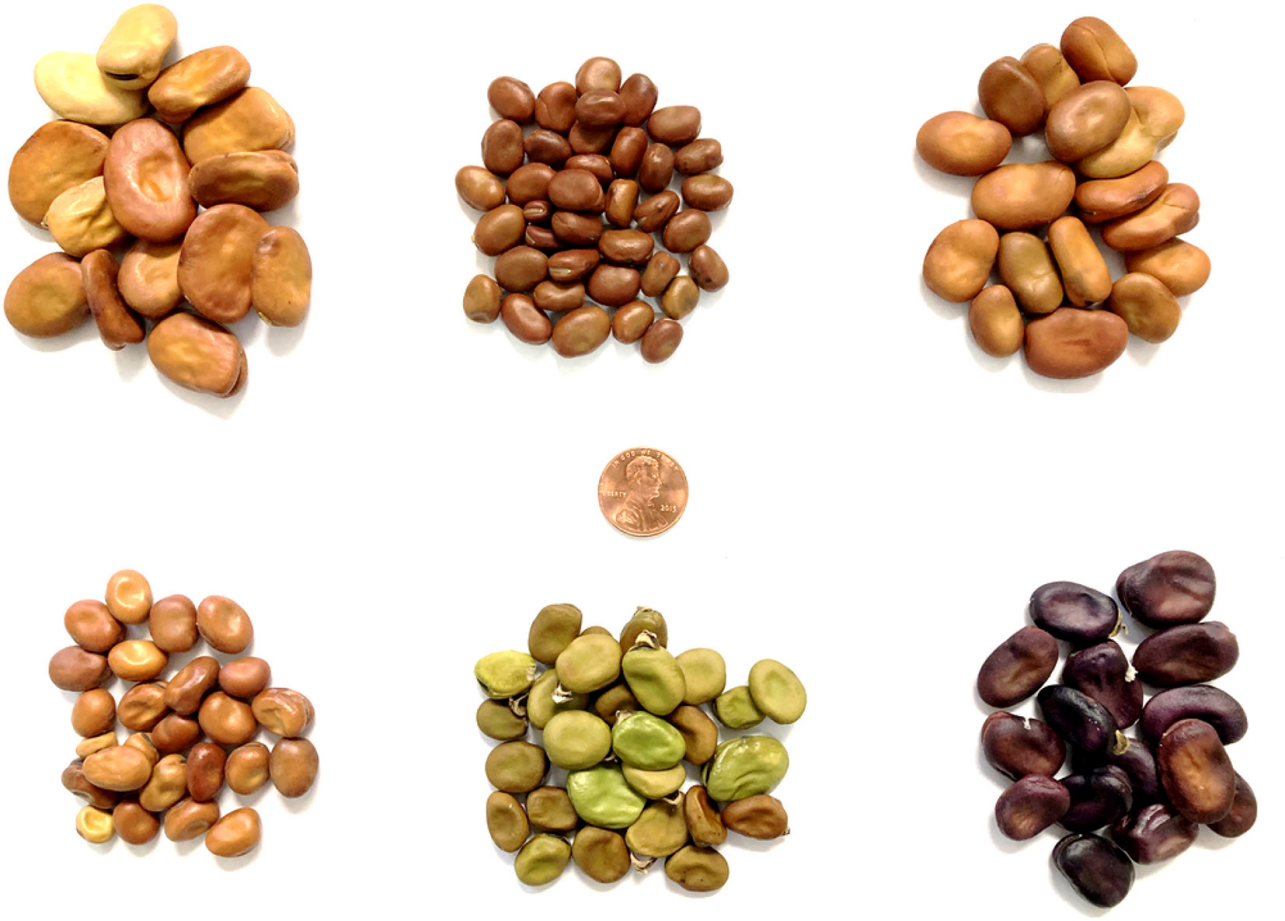
Faba bean seed varieties used in the study. Top row (left to right): Windsor, EN3, Aprovecho. Bottom row (left to right): Ziyad, EN47, Grano.

**FIGURE 2:**
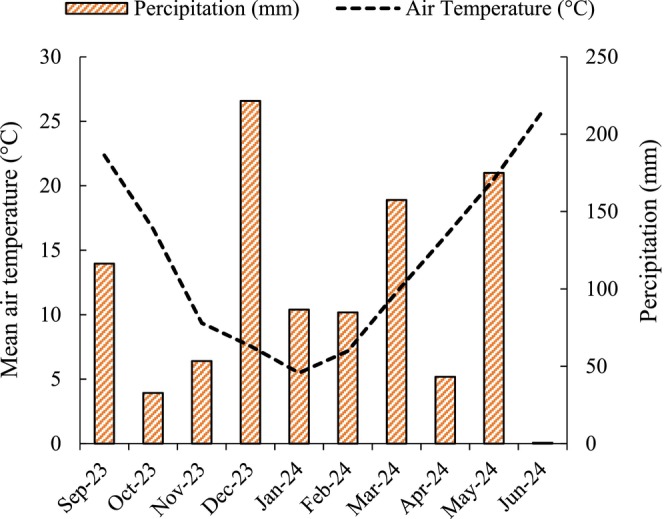
Mean monthly air temperature and total rainfall during the 2023–2024 growing season at Virginia State University's Randolph Farm, Petersburg, Virginia.

The experiment was conducted in 2023 and 2024 at Randolph Farm, the Virginia State University Research and Extension facility located in Chesterfield County, Virginia (37°13′43″ N; 77°26′2″ W). The objective was to assess the nutritional performance of six faba bean ( *Vicia faba* L.) varieties: ‘Grano’, ‘Ziyad’, ‘Aprovecho’, ‘EN3’, ‘EN47’, and ‘Windsor’ (Figure 1) under different planting dates. Detailed plant introduction numbers for each variety are provided in Table 1. A factorial randomized complete block design (RCBD) with three replications was implemented. Two planting dates were tested: September 22, 2023 (fall planting) and February 29, 2024 (spring planting). Figure 2 shows the mean air temperature and total rainfall recorded from September 2023 to June 2024 at Virginia State University's Randolph Farm, Petersburg, Virginia. Before planting, the soil was tilled using a disk harrow to achieve a fine, uniform seedbed. Soil samples were collected to determine baseline fertility levels, with results summarized in Table 2. Three raised beds (60 cm wide) were established to represent three replications. Varieties were sown on raised beds at 15 cm intra‐row spacing along 3‐m‐long plots. A 1‐m buffer was maintained between plots.

**TABLE 1:** Description of faba bean varieties planted at the Research and Extension Randolph Farm, Virginia State University.

Genotype	GRIN USA plant introduction
Windsor	—
EN47	PI 568235
EN3	PI 254006
Grano	PI 655347
Ziyad	PI 655348
Aprovecho	—

**TABLE 2:** Soil chemical properties at Randolph Farm based on pre‐planting analysis.

CEC	pH	Acidity	Base saturation	P	K	Ca	Mg	Zn	Mn	Cu	Fe	B
meq/100 g		%	%	mg kg^−1^				ppm				
2	6.4	2	98	56	56	314	25	0.4	4.9	0.3	25.1	0.1

No irrigation or seed inoculation was used throughout the trial. However, all seeds were treated with Vibrance Maxx (Syngenta US) at a rate of 0.1 mL per 100 g of seed to protect against seedborne and soilborne pathogens. Pre‐emergent herbicides, including Treflan (trifluralin) and Dual II Magnum (S‐metolachlor), were applied at a rate of 1100 mL ha^−1^ to control annual grasses and small‐seeded broadleaf weeds. To manage soilborne diseases, Ridomil Gold EC (Syngenta) was applied pre‐planting at the same rate. During the growing season, Priaxor fungicide was applied as a foliar spray at a rate of 2 fl oz per acre twice, once at the seedling stage and at the flowering stage. Weeds were controlled manually throughout the season. Once plants were established, fertilizers were applied by hand across all plots, including 40 kg ha^−1^ N (as urea), 30 kg ha^−1^ P (P_2_O_5_), and 40 kg ha^−1^ K (as K_2_O).

Plants sown in September 2023 were harvested in mid‐May 2024 (average growth period: 240 days), while those sown in February 2024 were harvested in early June 2024 (average growth period: 115 days). At maturity, dried pods were collected; if not fully dry, they were further air‐dried in a warm room. Seeds were then removed, labeled, and sent to Element Materials Technology Laboratory in Portland, Oregon (USA) for nutritional analysis. Ash content was analyzed following the official AOAC method 923.03 using a Thermo Fisher Thermolyne Muffle Furnace (Model F30430CM). Fat content was determined by the AOAC acid hydrolysis method 922.06. Protein concentration was measured using the AOAC combustion method 990.03 with a Leco FP928 Nitrogen and Protein Analyzer. Moisture and solids were quantified according to AOAC method 925.10. Mineral concentrations, including calcium (Ca), magnesium (Mg), potassium (K), phosphorus (P), sodium (Na), iron (Fe), and zinc (Zn), were measured using Inductively Coupled Plasma Mass Spectrometry (ICP‐MS) following EPA Method 6020B. Samples were prepared by microwave digestion with nitric acid using an Anton Paar Multiwave Go Plus system. Elemental analysis was performed with an Agilent 7700 Series ICP‐MS (Model G3281A) equipped with an ASX‐500 autosampler. Caloric and carbohydrate contents were calculated based on standard conversion factors derived from nutrient data. To better evaluate the nutritional quality of faba bean seeds, several derived parameters were calculated based on the measured values of protein, fat, carbohydrate, and caloric content. The following nutritional ratios were computed (Schakel et al. 2009; Friedman 1996):

$$\begin{array}{c} \text{Protein carbohydrate ratio}\;\left(\text{Protein}/\text{Carb}\right)=\\ \frac{\text{Protein content}\;\left(\%\right)}{\text{Carbohydrate content}\;\left(\%\right)} \end{array}$$

$$\begin{array}{c} \text{Protein calorie ratio}\;\left(\text{Protein}/\text{Calories}\right)=\\ \frac{\text{Protein content}\;\left(g/100g\right)\times 4}{\text{Total energy}\;\left(\text{kcal}/100g\right)} \end{array}$$

$$\mathrm{Fat}\;\text{calorie ratio}\;\left(\mathrm{Fat}/\text{Calories}\right)=\frac{\mathrm{Fat}\;\text{content}\;\left(g/100g\right)\times 9}{\text{Total energy}\;\left(\text{kcal}/100g\right)}$$

$$\begin{array}{c} \text{Carbohydrate calorie ratio}\;\left(\text{Carb}/\text{Calories}\right)=\\ \frac{\text{Carbohydrate content}\;\left(g/100g\right)\times 4}{\text{Total energy}\;\left(\text{kcal}/100g\right)} \end{array}$$

$$\text{Protein energy ratio}\;\left(\mathrm{PE}\;\text{ratio}\right)=\frac{\text{Protein content}\;\left(g/100g\right)}{\text{Total energy}\;\left(\text{kcal}/100g\right)}\times 1000$$

For nutrient density calculations, the concentration of each mineral nutrient (e.g., Ca, K, P, Mg, Fe, Na, Zn) was normalized per unit of energy to reflect its contribution per calorie (Drewnowski 2009):

$$\begin{array}{c} \text{Nutrient density}\;\left(\mathrm{mg}/100\;\text{kcal}\right)=\\ \frac{\text{Nutrient concentration}\;\left(\mathrm{mg}/100g\right)}{\text{Total energy}\;\left(\text{kcal}/100g\right)}\times 100 \end{array}$$

Statistical analysis was conducted using SAS 9.4 (SAS Institute Inc., Cary, NC, USA). A two‐factor ANOVA was performed to assess the effects of variety, planting date, and their interaction. Mean comparisons were made using the Least Significant Difference (LSD) test at a significance level of *p* ≤ 0.05. A Principal Component Analysis (PCA) was carried out using R Studio software (version 4.4.1), including data from all varieties to examine the relationships among seed nutritional traits such as calories, protein, fat, carbohydrate, nutrient concentrations, and their ratios.

## 3 Results

### 3.1 Calories, Protein, Fat, Carbohydrate, and Ratios

**FIGURE 3:**
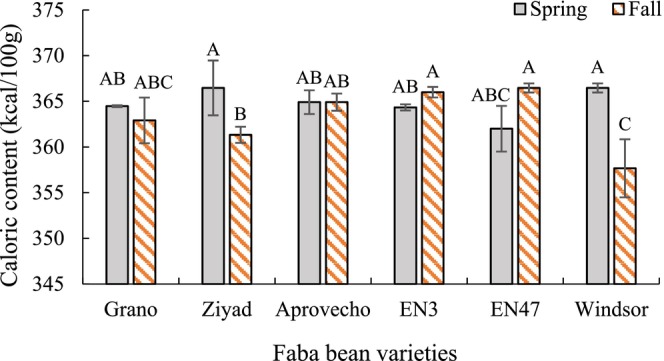
Interaction effects of variety and planting date on the caloric content of faba bean seeds. Different letters indicate significant differences according to the least significant difference (LSD) test at *p* < 0.05. Bars represent means ± standard error.

The mean values of calories, ash, protein, fat, carbohydrate, and associated nutritional ratios are presented in Table 3. Variety and planting date did not have a significant effect on calorie content, fat, or the fat calorie ratio. However, the interaction between variety and planting date was significant for calorie content (Figure 3). Except for the varieties Ziyad and Windsor, no significant differences in seed calorie content were observed between planting dates. In both Ziyad and Windsor, calorie content decreased when planted in the fall compared to the spring.

**TABLE 3:** Mean values of calories, protein, fat, carbohydrate, and ratios in faba bean varieties planted in fall and spring.

	Calories	Ash	Protein	Fat	Carb	Protein/Carb	Protein/Calories	Fat/Calories	Carb/Calories	PE ratio
	kcal/100 g	%	%	%	%	%	%	%	%	mg/kcal
Variety										
Grano	363	4.23B	20.0C	1.96	66.2A	30.4C	55.2C	4.87	73.0A	22.1C
Ziyad	363	4.18 BC	26.3A	1.70	60.6C	43.4A	72.5A	4.21	66.7C	29.0A
Aprovecho	364	4.50A	23.4AB	2.35	62.3 BC	38.0AB	64.2AB	5.81	68.4 BC	25.7AB
EN3	365	3.81D	21.6 BC	2.03	65.0AB	33.7 BC	59.3 BC	5.01	71.2AB	23.7 BC
EN47	363	4.10 BC	21.2 BC	2.02	65.0AB	32.9 BC	58.4 BC	4.99	71.6AB	23.3 BC
Windsor	360	4.02 DC	20.2C	1.84	65.8A	30.8C	56.0 BC	4.59	72.9A	22.4 BC
Planting date										
Spring	364	4.05	23.7A	1.98	62.7B	38.0A	65.3A	4.90	68.9B	26.1A
Fall	362	4.10	20.5B	1.90	65.8A	31.6B	56.6B	4.73	72.6A	22.6B

*Note:* Different letters within columns in each parameter indicate significant differences by the least significant difference (LSD) test at *p* < 0.05. Carb: carbohydrate; protein‐to‐energy ratio: PE ratio.

**FIGURE 4:**
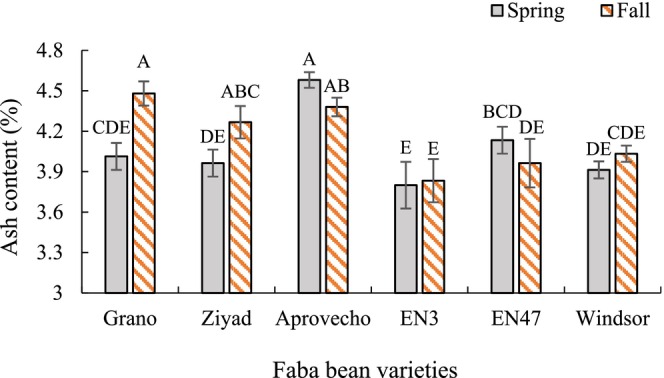
Interaction effects of variety and planting date on the ash content of faba bean seeds. Different letters indicate significant differences according to the least significant difference (LSD) test at *p* < 0.05. Bars represent means ± standard error.

Across planting dates, Aprovecho exhibited the highest ash content (4.5%), while Windsor had the lowest (4.02%) (Table 3). Although planting date alone did not significantly affect ash content, the interaction between planting date and variety was significant (Figure 4). Ash content increased by 7% and 11% in fall‐planted Ziyad and Grano, respectively, compared to their spring counterparts. Other varieties did not show significant differences in ash content between planting dates.

Variety significantly influenced the protein content (Table 3). Ziyad had the highest protein content at 26.3%, followed by Aprovecho at 23.4%. The lowest protein levels were recorded in Grano and Windsor, at 20.0% and 20.2%, respectively. Planting date had a significant effect on protein content, with spring‐planted faba beans exhibiting 15% higher protein levels than those planted in the fall (Table 3).

**FIGURE 5:**
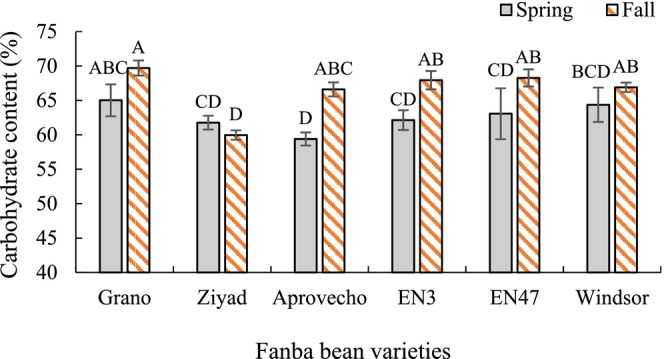
Interaction effects of variety and planting date on the carbohydrate content of faba bean seeds. Different letters indicate significant differences according to the least significant difference (LSD) test at *p* < 0.05. Bars represent means ± standard error.

Carbohydrate content was significantly influenced by planting date, variety, and their interaction. Across planting dates, Grano and Windsor had the highest carbohydrate contents at 66.2% and 65.8%, respectively, with no significant difference compared to EN47 and EN3 (60.0%). Ziyad had the lowest carbohydrate content at 60.6% (Table 3). Among the varieties, the carbohydrate content of Aprovecho, EN3, and EN47 increased significantly when planted in fall, whereas the other varieties showed no significant change. The most notable increase was observed in Aprovecho, with a 12% rise under fall planting (Figure 5).

Both planting date and variety significantly influenced the protein‐to‐carbohydrate and protein‐to‐calorie ratios (Table 3). The highest ratios were observed in Ziyad, followed by Aprovecho. These ratios decreased by 16% and 13%, respectively, under fall planting compared to spring. The carbohydrate‐to‐calorie ratio was also significantly affected by planting date and variety. Grano and Windsor exhibited the highest carbohydrate calorie ratios at 73.0% and 72.9%, respectively, which were not significantly different from EN47 and EN3. Ziyad had the lowest carbohydrate calorie ratios at 66.7%. Planting in the fall increased the carbohydrate calorie ratio by approximately 5% (Table 3).

The PE ratio was significantly affected by both planting date and variety (Table 3). Among all varieties, Ziyad had the highest PE ratio at 29 mg/kcal, showing no significant difference from Aprovecho, while Grano had the lowest at 22.1 mg/kcal. Seeds planted in spring had a 15% higher PE ratio than those planted in fall.

### 3.2 Nutrient Content of Faba Bean

The results presented in Table 4 show that variety had a significant effect on potassium (K), K density, phosphorus (P), P density, sodium (Na), and Na density. However, no significant varietal effects were observed for calcium (Ca), iron (Fe), magnesium (Mg), zinc (Zn), or their respective densities. Grano had the highest K content at 1580 mg/100 g and the highest K density at 453 mg/100 kcal, followed by Aprovecho with 1540 mg/100 g and 423 mg/100 kcal, respectively. In contrast, EN3 had the lowest K content (1260 mg/100 g) and the lowest K density (345 mg/100 kcal). For P, the highest concentrations were observed in Aprovecho (755 mg/100 g) and Ziyad (750 mg/100 g), followed by Grano (730 mg/100 g). The lowest values were recorded in EN47 and EN3, with 624 mg/100 g and 625 mg/100 g, respectively. A similar trend was observed for P density, with Aprovecho and Ziyad showing the highest values among all varieties (Table 4). In terms of Na content, EN47 and Aprovecho had the highest values at 2.17 and 2.02 mg/100 g, respectively. The remaining varieties did not differ significantly from each other. Na density followed the same trend, with EN47 and Aprovecho exhibiting the highest values at 0.59 and 0.55 mg/100 kcal, respectively (Table 4).

**TABLE 4:** Mean values of nutrient content and nutrient density in faba bean varieties planted in fall and spring.

	Ca	Fe	K	Mg	P	Zn	Na	Ca density	Fe density	K density	Mg density	P density	Zn density	Na density
	mg/100 g							mg/100 kcal						
Variety														
Grano	126	5.57	1580A	158	730AB	3.60	1.03B	34.7	1.53	435A	43.5	201AB	0.99	0.28B
Ziyad	132	5.27	1360CD	142	750A	4.20	0.75B	36.4	1.45	374 DC	39.2	206A	1.15	0.20B
Aprovecho	145	4.67	1540AB	170	755A	4.15	2.02A	39.9	1.28	423AB	46.8	207A	1.14	0.55A
EN3	134	4.86	1260D	145	625C	3.15	1.00B	36.7	1.33	345D	39.9	171C	0.86	0.27B
EN47	128	4.88	1422 BC	149	624C	3.76	2.17A	35.3	1.34	391 BC	41.1	171C	1.03	0.59A
Windsor	150	4.99	1410 BC	159	657 BC	3.31	1.08B	41.8	1.38	390 BC	44.1	182 BC	0.91	0.30B
Planting date														
Spring	125B	5.28A	1432	159A	661	3.81	1.44A	34.3B	1.45A	393	43.6A	181	1.04	0.39A
Fall	146A	4.78B	1363	144B	693	3.45	1.11B	40.4A	1.32B	376	39.8B	191	0.95	0.30B

*Note:* Different letters within columns in each parameter indicate significant differences by the least significant difference (LSD) test at *p* < 0.05. Ca: calcium; Fe: iron; K: potassium; Mg: magnesium; P: phosphorus; Zn: zinc; Na: sodium.

Planting date significantly affected Ca, Fe, Mg, and Na content and their respective densities (Table 4). Seeds planted in the spring had significantly higher Fe, Mg, and Na contents by 10%, 10%, and 29%, respectively, compared to those planted in the fall. However, Ca content was 16% higher in fall‐planted seeds than in those planted in spring. Corresponding nutrient densities showed similar patterns, and Fe, Mg, and Na densities were significantly higher under spring planting, while Ca density was 17% higher under fall planting (Table 4).

### 3.3 Principal Component Analysis

**FIGURE 6:**
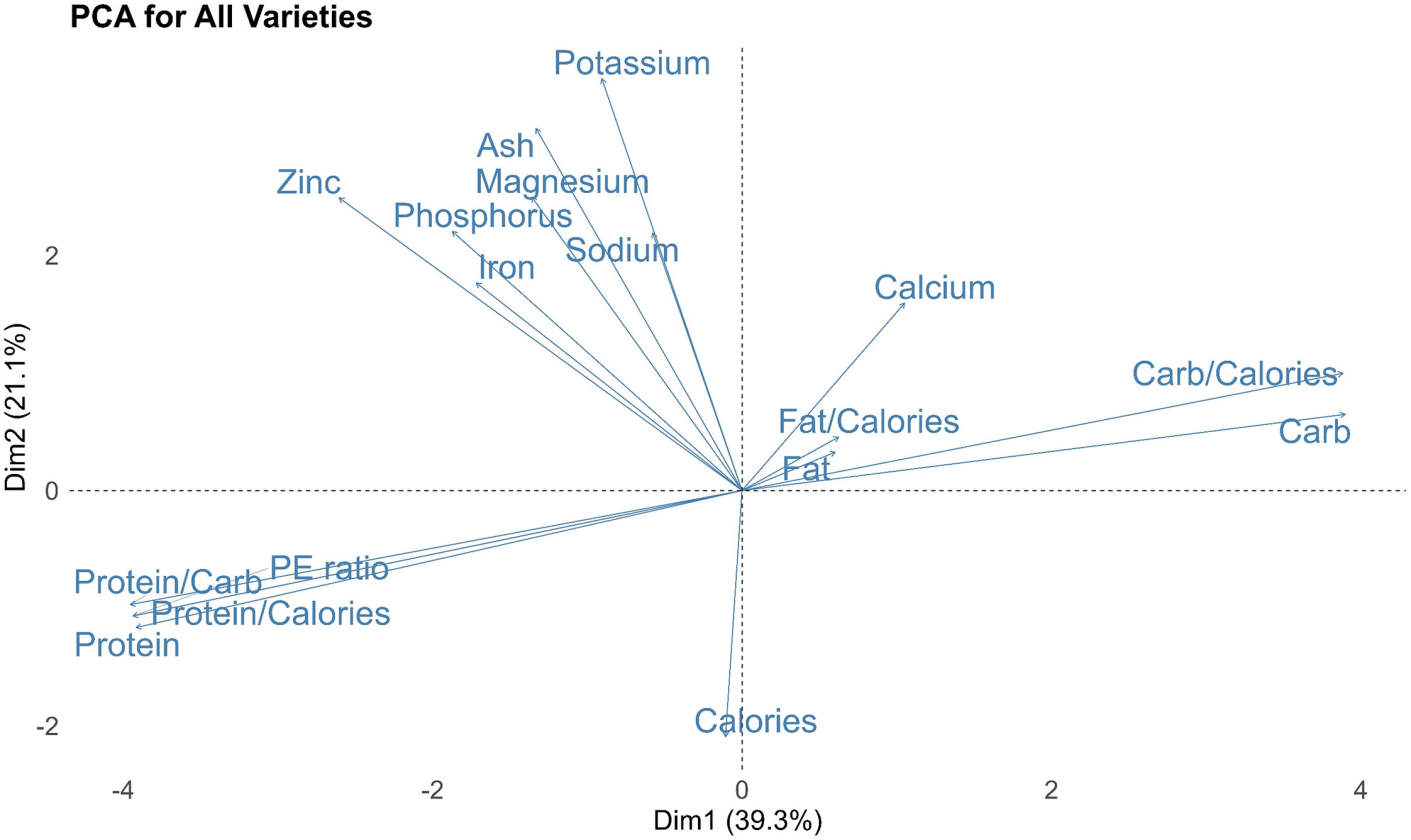
Principal component analysis (PCA) of seed nutritional traits, including calories, protein, fat, carbohydrate, their ratios, and nutrient concentrations.

Principal Component Analysis (PCA) was conducted to explore the relationships among seed nutritional traits, including calories, protein, fat, carbohydrate, nutrient concentrations, and their derived ratios (Figure 6). The PCA biplot reveals that Ca, K, Na, Mg, P, Fe, Zn, and ash clustered closely and had strong correlations among these mineral nutrients. The protein‐to‐calorie ratio, protein‐to‐carbohydrate ratio, and PE ratio were strongly aligned with the protein vector. Similarly, the carbohydrate‐to‐calorie ratio was positively correlated with carbohydrate content. PCA revealed a strong negative correlation between protein and carbohydrate. The calorie vector pointed in a direction opposite to that of most mineral nutrients, indicating a negative correlation between total caloric content and nutrient content. Both fat and fat‐to‐calorie ratio had relatively short vectors, suggesting that these traits were weakly correlated with other traits in the dataset (Figure 6).

## 4 Discussion

We highlight our contribution by showing that uncharacterized faba bean varieties respond in seed composition to swing dates, which is not documented in the current literature. The study shows that both variety and planting date have a significant influence on the nutritional composition of faba bean seeds. While calorie and fat contents remained relatively stable across planting dates, notable differences were observed in protein, carbohydrate, and derived nutritional ratios. Several studies have demonstrated that faba bean varieties differ significantly in their seed composition (Baloch et al. 2014; Khazaei and Vandenberg 2020; Warsame et al. 2020; Hacisalihoglu 2024). Among the six faba bean varieties evaluated, Ziyad had the highest protein content (26.3%) and the lowest carbohydrate content. It also had the highest protein‐to‐calorie, protein‐to‐carbohydrate, and PE ratios, indicating a greater contribution of protein per unit of energy. In contrast, Windsor and Grano, which had the lowest protein contents, showed the highest carbohydrate levels, suggesting a greater energy contribution from carbohydrates. Aprovecho ranked second after Ziyad in protein content and nutritional ratios. An inverse relationship often exists between seed protein and carbohydrate contents. Genotypes with higher protein levels typically show greater amino acid accumulation during seed development, indicating enhanced nitrogen assimilation and amino acid uptake (Golombek et al. 2001). Ash content also varied among varieties, with Aprovecho showing the highest and Windsor the lowest levels.

Significant varietal differences were observed in several nutritional parameters of faba bean seeds, particularly in K, P, Na, and their respective nutrient densities (Table 4). No significant differences were found among varieties for Ca, Fe, Mg, Zn, or their densities. Among the varieties, Grano consistently ranked high in K‐related traits, exhibiting the highest K content (1580 mg/100 g) and highest K density (453 mg/100 kcal). Aprovecho followed closely with 1540 mg/100 g and 423 mg/100 kcal, respectively. For P, Aprovecho and Ziyad demonstrated the highest P content (755 and 750 mg/100 g, respectively) and the highest P densities. Grano also showed high P content (730 mg/100 g). In terms of Na, EN47 and Aprovecho stood out with the highest Na content (2.17 and 2.02 mg/100 g, respectively) and Na densities (0.59 and 0.55 mg/100 kcal). The remaining varieties showed no significant differences in Na content or density.

Planting date had a marked effect on protein, carbohydrate, and their derived ratios (Table 3). However, no significant effects were observed on calorie, ash, or fat contents. Spring planting led to higher protein concentrations, approximately 15% more than fall planting, and improved nutritional indices such as the protein‐to‐calorie and protein‐to‐carbohydrate ratios, as well as the PE ratio. Planting date significantly influences the protein and carbohydrate content in legume seeds by affecting the growing season length and environmental conditions during seed development (Bellaloui et al. 2011; Hu and Wiatrak 2012). Results showing the effect of planting date on protein content were not consistent. Studies on soybean show that early planting under irrigated conditions increased seed oil and oleic acid, but decreased protein compared to late planting (Bellaloui et al. 2011). However, Kumar et al. (2006) indicated that shortened duration from flowering to maturity might have contributed to the reduction of protein accumulation in soybean. The concentration of essential amino acids in tepary bean grains can also be significantly changed by sowing date (Ghadimian et al. 2020). In faba beans, temperature did not directly influence the content of starch, protein, and low molecular weight carbohydrates, but their accumulation followed the seed moisture content (Lundby et al. 2024).

While spring planting has often been associated with higher seed protein concentrations in some crops compared to fall planting, the cited studies do not provide a detailed mechanistic explanation for this phenomenon (Bagherikia et al. 2025; Nuttall et al. 2017). However, insights from various studies suggest that environmental conditions, nutrient availability, and plant physiology may indirectly explain this trend. Factors such as temperature, day length, and moisture availability, which differ between spring and fall plantings, can influence protein accumulation in seeds (Salmerón et al. 2022; Xu et al. 2022), affecting plant development and nutrient partitioning. Additionally, the timing of nutrient uptake and remobilization can vary between spring and fall plantings, impacting seed protein content (Martre et al. 2003). Nitrogen, as a key component of protein, requires adequate availability and efficient translocation to developing seeds (Martre et al. 2003). Spring planting may coincide with more favorable conditions for nitrogen uptake and assimilation during critical growth stages, thereby leading to higher protein concentrations in seeds (Subedi et al. 2007).

The fall planting resulted in a significant increase in carbohydrate content across all varieties except Ziyad. The carbohydrate‐to‐calorie ratio was also higher by approximately 5% under fall conditions. Although ash content was not significantly affected by planting date alone, significant interactions were observed between variety and planting date. For example, Ziyad and Grano showed increased ash content under fall planting. The PCA revealed an inverse correlation between protein and carbohydrate contents (Figure 6). This relationship in seeds is governed by complex metabolic and regulatory mechanisms, including assimilate partitioning and responses to environmental cues (Weber et al. 2005). High temperatures are known to shorten the seed‐filling period (Chimenti et al. 2001). Spring planting resulted in a shorter vegetative period for faba bean, which may have contributed to a significant reduction in seed carbohydrate content. This outcome suggests a metabolic trade‐off where the plant prioritizes the synthesis and accumulation of proteins at the expense of carbohydrates when the growing season is truncated (Wingler and Soualiou 2025). Similarly, fall‐planted barley exhibited higher grain yield and malt extract than spring‐planted crops (Saygili 2023). This suggests that earlier fall plantings allow for more established plants with potentially greater carbohydrate resources. The fat content of faba bean seeds did not vary with planting date, likely due to inherent regulatory mechanisms that stabilize fatty acid synthesis against environmental changes. Certain species maintain consistent fatty acid profiles through robust enzymatic control and metabolic regulation (Voelker and Kinney 2001). Similar stability has been observed in flax, where fat concentration remained unaffected by seeding date (Siddique and Wright 2004).

In our study, the PCA showed that fat had a short vector and was oriented in the opposite direction to protein, indicating a negative correlation (Figure 6). It is well established that oil and protein concentrations are negatively correlated, a relationship influenced not only by genetic variation but also by environmental factors (Song et al. 2016). The inverse correlations are a consequence of the competition for carbon and nitrogen sources during seed development. As a plant allocates more carbon to lipid (oil) biosynthesis, less is available for amino acid and protein synthesis, and vice versa (Pipolo et al. 2004; Allen and Young 2013).

Planting date had a significant influence on the mineral nutrient composition of faba bean seeds. Seeds planted in the spring exhibited significantly higher concentrations of Fe, Mg, and Na compared to those planted in the fall. Specifically, Fe, Mg, and Na contents increased by approximately 10%, 10%, and 29%, respectively, under spring conditions. This trend was consistent in nutrient density values. The increased uptake and accumulation of Fe and Mg in spring‐planted seeds may be attributed to more favorable soil temperature and moisture conditions during key vegetative and reproductive stages. In contrast, Ca content and density were significantly higher under fall planting, with an average increase of 16% and 17%, respectively, compared to spring planting. Planting date influences soil nutrient availability, uptake efficiency, and nutrient translocation from leaves to seeds by shaping environmental conditions, root–microbe interactions, and physiological processes during seed development (Bellaloui et al. 2011; Mondal and Bose 2019). In soybeans, early planting under irrigated conditions resulted in lower seed Fe concentrations compared to late planting (Bellaloui et al. 2011), which aligns with our findings. Magnesium uptake and transport rates by barley seedlings were found to be lower at root temperatures of 5°C and 15°C compared to 25°C (Schimansky 1981). Similar to Mg, microbial Fe (III) reduction in wetland soils increases during warmer summer conditions, with maximum rates significantly higher at 18°C than at 6°C or 12°C (Schilling et al. 2019). Research on Sierra Nevada riparian soil showed that resin‐available Na^+^ and K^+^ varied significantly by season (winter vs. summer and fall) (Blank 2010). Sorption characteristics for both ions were temperature‐dependent, with the proportional sorption of K^+^ significantly reduced at 1°C (Blank 2010). The elevated Ca levels in fall‐planted seeds may be attributed to the longer growing season during seed development. Given the farm's soil pH of 6.4, which is suboptimal for Ca availability, a prolonged growth period likely provided plants with more time and opportunity to absorb and translocate Ca (Nistor et al. 2022). The PCA biplot shows that Ca, K, Na, Mg, P, Fe, and Zn cluster closely, indicating strong positive correlations among these mineral nutrients (Figure 6). The strong correlations observed among Ca, K, Na, Mg, P, Fe, and Zn in seeds likely reflect shared physiological and biochemical pathways regulating their uptake, transport, and storage. These nutrients may be co‐transported through shared membrane transporters and loaded into the phloem via similar energy‐dependent mechanisms (White and Broadley 2003; Marschner 2012). In soybean seeds, positive correlations have been reported between Zn, P, and S, as well as between Zn, Cu, selenium (Se), and rubidium (Rb) (Hacisalihoglu and Settles 2017). In another study on common bean, seed Fe and Zn were significantly correlated with each other (Akond et al. 2011).

## 5 Conclusion

This study demonstrated that both planting date and variety significantly influenced the nutritional composition of faba bean seeds. While calorie and fat contents were generally stable across planting dates and varieties, protein, carbohydrate, ash, and several nutrient concentrations varied notably. Spring planting enhanced protein content, PE ratio, and levels of Fe, Mg, and Na, whereas fall planting increased carbohydrate concentration and Ca content. Among the varieties, Ziyad consistently showed the highest protein content, while Grano and Windsor were highest in carbohydrate content and carbohydrate‐derived energy. Mineral nutrients such as K, P, and Na also varied across genotypes, with Grano and Aprovecho showing higher K and P levels, while EN3 and EN47 were generally lower. PCA analysis revealed strong correlations among mineral nutrients and distinct inverse relationships between protein and carbohydrate. As a recommendation, further studies are needed to investigate the influence of sowing date on detailed seed composition traits, particularly amino acid profiles, starch and sugar content, fatty acid composition, vitamin levels, and antinutritional compounds such as vicin.

## Funding

This work is supported by the Research Capacity Fund (Evans‐Allen program), project award no. 7004952 and the Virginia Department of Agriculture and Consumer Services, Specialty Crop Block Grant Program, project award no. 2024‐592, from the U.S. Department of Agriculture's National Institute of Food and Agriculture.

## Conflicts of Interest

The authors declare no conflicts of interest.

## Supporting information


**Data S1:** pei370117‐sup‐0001‐DataS1.xlsx.

## Data Availability

The data that support the findings of this study are available in the Supporting Information of this article.
